# Nomogram for predicting overall survival of metastatic pancreatic cancer patients based on HBV infection and inflammatory-nutritional biomarkers

**DOI:** 10.3389/fonc.2024.1362566

**Published:** 2024-07-04

**Authors:** Xiawei Long, Qian Li, Sina Liao, Youzhi Lin, Xiaoli Liao

**Affiliations:** ^1^ Department of First Chemotherapy, Guangxi Medical University Cancer Hospital, Nanning, China; ^2^ Hepatobiliary Surgery Department, Guangxi Medical University Cancer Hospital, Nanning, China

**Keywords:** metastatic pancreatic cancer, hepatitis B virus, systemic inflammation, prognostic nutritional index, nomogram, overall survival

## Abstract

**Purpose:**

To develop and validate a nomogram for predicting the overall survival of patients with metastatic pancreatic cancer.

**Methods:**

This retrospective study included 236 patients with metastatic pancreatic cancer treated at Guangxi Medical University Cancer Hospital between October 2013 and October 2022. Patients were grouped according to hepatitis B virus (HBV) infection status. Cox proportional hazard regression was used to identify the prognostic factors independently associated with overall survival. Results were used to build a nomogram, which was assessed through internal validation using bootstrap resampling.

**Results:**

Patients in the HBV-positive group (N = 37) showed significantly better overall survival than those in the HBV-negative group (N=199; *P* = 0.014). Overall survival was independently associated with the following factors: HBV infection status, sex, chemotherapy, metastatic sites, a combined index of hemoglobin, albumin, lymphocytes, and platelets, neutrophil-albumin ratio, as well as levels of CA125. The nomogram showed good predictive power, with an area under the curve of 0.808 for the time-dependent receiver operating characteristic. Calibration and decision curve analyses indicated good calibration and clinical usefulness of the nomogram for predicting the overall survival of patients with metastatic pancreatic cancer.

**Conclusion:**

A nomogram based on the HBV infection status and inflammatory nutritional markers may help predict the overall survival of patients with metastatic pancreatic cancer and guide personalized clinical treatment.

## 1 Introduction

Pancreatic cancer is highly aggressive, with a 5-year overall survival (OS) of only 12%, making it the third leading cause of cancer-related mortality in the United States (1). Globally, the incidence of pancreatic cancer in 2020 was estimated to be 495,773 (2). The poor prognosis of pancreatic cancer is mainly due to most patients being diagnosed at an advanced metastatic stage. The median OS for metastatic pancreatic cancer (mPC) is only 3 months (3). Identifying prognostic factors that predict worse OS among patients with mPC could help personalize treatments and potentially extend lifespan.

One potential determinant of OS in patients with mPC is chronic infection with the hepatitis B virus (HBV) (4–8). Despite widespread vaccination programs, over 250 million people worldwide are infected with HBV (9). Chronic HBV infection is associated with a high risk of pancreatic and other cancers (10, 11). HBV infection may increase cancer risk by inducing unchecked inflammatory responses (11, 12). Several inflammatory biomarkers and nutritional indicators, such as the neutrophil-to-albumin ratio (NAR) and the combined index of hemoglobin, albumin, lymphocytes, and platelets (HALP), correlate with prognosis in many cancers (13–16). However, the predictive values of these biomarkers for mPC remain unclear.

In this study, we constructed a nomogram based on HBV infection status, inflammatory and nutritional indicators to predict the prognosis of patients with mPC and provide personalized comprehensive treatment.

## 2 Materials and methods

### 2.1 Patients

This retrospective study was approved by the Ethics Review Committee of Guangxi Medical University Cancer Hospital (IRB approval number: LW2023006), which waived the requirement for informed patient consent because the patients had previously provided written consent at the time of treatment for their anonymized medical data to be analyzed and published for research purposes.

**Figure 1:**
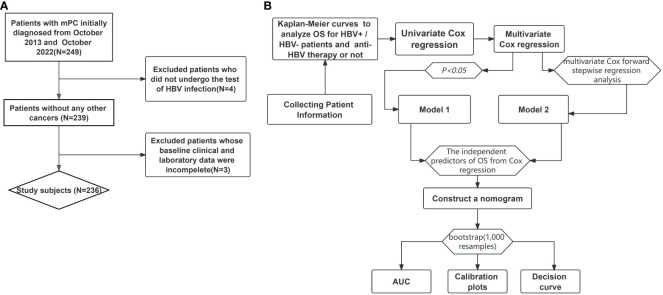
Flow chart of mPC patient selection process **(A)** and statistical flow chart **(B)**.

We screened consecutive patients diagnosed with mPC and treated at Guangxi Medical University Cancer Hospital between October 2013 and October 2022 (**Figure 1A**). Patients were enrolled if they (1) were older than 18 years, (2) had histologically or cytologically confirmed mPC, and (3) were assigned stage IV based on the 8th edition of the tumor-node-metastasis staging system developed by the American Joint Committee on Cancer. Patients were excluded if they (1) had other malignant comorbidities, (2) had not been tested for HBV infection at our hospital, or (3) had incomplete clinical data.

### 2.2 Data collection

Baseline clinicodemographic data, including age, sex, T stage, N stage, number of metastatic locations, diabetes, primary tumor location, and details of chemotherapy, radiotherapy, surgery, and anti-HBV treatment, were extracted from the electronic medical records of our hospital. Blood samples were collected from each patient during their initial mPC diagnosis. These samples were analyzed to determine neutrophils, lymphocytes, and platelets counts, hemoglobin, albumin, CA125, and CA19–9 levels, and the presence of HBV antigen, anti-HBV antibodies, and HBV DNA. The NAR was calculated as the ratio of neutrophil count (10^9^/L) to albumin concentration (g/L) (13). HALP was calculated as hemoglobin level (g/L) × albumin level (g/L) × lymphocyte count (10^9^/L)/platelet count (10^9^/L) (17).

An enzyme-linked immunosorbent assay was used to screen for hepatitis B surface antigen (HBsAg), hepatitis B envelop antigen (HBeAg) and their associated antibodies, as well as for the hepatitis B core antibody (anti-HBc). HBV DNA was detected by polymerase chain reaction. Quality controls included the timeliness and the selection of positive and negative control sera.

Patients who tested positive for HBsAg and either HBeAg or HBV DNA were classified as HBV-positive, otherwise, they were classified as HBV-negative. These two groups were compared in terms of the blood indices mentioned above and in terms of OS, defined as the period between mPC diagnosis and death, loss to follow-up, or completion of the last follow-up on April 30, 2024. Survival data were extracted from medical records and telephone follow-ups.

### 2.3 Statistical analysis

All statistical analyses were performed using SPSS (version 25.0, IBM SPSS, Inc., Chicago, IL, USA), PASS 11.0(Power Analysis and Sample Size), and R 4.2.1 (http://www.r-project.org/). Results were statistically significant when associated with a two-sided *P* < 0.05. Differences between the HBV-positive and -negative groups in continuous variables were assessed for significance using the Student’s *t* test or Wilcoxon test. Differences in categorical variables were evaluated using the chi-squared test or Fisher’s exact test. Continuous variables were reported as medians with interquartile ranges, while categorical data were expressed as whole numbers and proportions. NAR and HALP were calculated as continuous variables but categorized as “low” or “high” using a cut-off value that optimally separated patients based on OS in X-tile (version 3.6.1).

The PASS 11.0 software was used to estimate the sample size at a level of the single side 0.05. OS was analyzed using Kaplan-Meier curves and compared between HBV-positive and HBV-negative patients using the log-rank test (**Figure 1B**). The OS of the patients who received anti-HBV therapy was also analyzed using the log-rank test. Univariate Cox regression was used to identify the characteristics associated with OS. Variables with *P <*0.05 in the univariate analysis were included in the multivariate Cox proportional hazards regression and were set as Model 1. Considering the interaction of multiple factors, Model 2 was also set for our multivariate Cox regression analysis, using the multivariate Cox forward stepwise regression analysis. Only one variable was included at each step. Variables with *P <*0.05 were included in the model, those with *P >*0.05 were discarded, and the next variable was included. The factors that were less important to the OS results were gradually eliminated. The final model provided a better prediction of the OS of the patients. Data were expressed as hazard ratios (*HRs*) and 95% confidence intervals (*CIs*).

Independent predictors of OS identified using Cox regression were used to construct a nomogram. The predictive power of the nomogram model was evaluated by the area under the curve (AUC) of the receiver operating characteristic using the “timeROC” in the R software package. An AUC greater than 0.7 indicated good performance of the nomogram model. Calibration plots were generated to assess the performance of the nomogram between the predicted probability and actual survival rate. The predictive power of the nomogram was internally validated using bootstrapping (1,000 resamples).

The bootstrap method involved resampling data from the original sample to create new samples. Resampling means that data can be sampled more than once. Based on these new samples, the required statistics were calculated to construct a calibration curve. In particular, we extracted data from 50 patients from a dataset of 236 patients to form a new dataset. We randomly sampled the data from one patient at a time from the original dataset and placed them in the new dataset. This process was repeated 1000 times to plot the calibration curve. The clinical benefit provided by the nomogram was assessed using decision curve analysis in the “ggDCA” package of the R software.

## 3 Results

### 3.1 Patient characteristics

Of the 249 patients considered for enrollment, 236 were included in the final analysis. The median age of these patients was 60.0 (52.0–68.0) years, and 135 (57.2%) were men (**Table 1**). The HBV-positive and HBV-negative groups comprised 37 and 199 patients, respectively. HBV-positive patients were significantly younger at mPC diagnosis than HBV-negative patients. The number of HBV-positive patients who were already taking anti-HBV drugs was 20 (54.05%). HBV-positive patients were treated with anti-HBV drugs before and until the end of chemotherapy. Some HBV-positive patients did not receive chemotherapy, such as those receiving only supportive care and those who refused to use anti-HBV drugs. Most tumors (60.59%) were found in the body and tail of the pancreas, while 84 (35.59%) were located in the head of the pancreas. No other significant differences were found in clinicopathological features between the two groups.

**Table 1:** Characteristics of mPC patients with HBV-positive group and HBV-negative group.

Variable	Total	HBV(+)	HBV(-)	*P*
	(n=236)	(n=37)	(n=199)	
**Age (median[*IQR*])**	60.0[52.0,68.00]	56.0[44.0,64.0]	61.0[54.0,69.0]	<0.001
Age (n,%)				
≤60	119 (50.42)	23 (62.16)	96 (48.24)	0.169
>60	117 (49.58)	14 (37.84)	103 (51.76)	
Sex(n,%)				
Male	135 (57.20)	22 (59.46)	113 (56.78)	0.9036
Female	101 (42.80)	15 (40.54)	86 (43.22)	
Tumor location(n,%)				
Head	84 (35.59)	17 (45.95)	67 (33.67)	0.1916
Body/Tail	143 (60.59)	20 (54.05)	123 (61.81)	
Unknown	9 (3.81)	0 (0.00)	9 (4.52)	
T stage(n,%)				
T1	3 (1.27)	0 (0.00)	3 (1.51)	0.9311
T2	33 (13.98)	6 (16.22)	27 (13.57)	
T3	57 (24.15)	8 (21.62)	49 (24.62)	
T4	125 (52.97)	20 (54.05)	105 (52.76)	
TX	18 (7.63)	3 (8.11)	15 (7.54)	
N stage(n,%)				
N0	43 (18.22)	8 (21.62)	35 (17.59)	0.7094
N1	116 (49.15)	16 (43.24)	100 (50.25)	
N2	65 (27.54)	10 (27.03)	55 (27.64)	
NX	12 (5.08)	3 (8.11)	9 (4.52)	
Metastatic sites(n,%)				
<3	173 (73.31)	30 (81.08)	143 (71.86)	0.336
≥3	63 (26.69)	7 (18.92)	56 (28.14)	
Chemotherapy(n,%)				
No	132 (55.93)	21 (56.76)	111 (55.78)	0.912
Yes	104 (44.07)	16 (43.24)	88 (44.22)	
Radiotherapy(n,%)				
No	228 (96.61)	37 (100.00)	191 (95.98)	0.4556
Yes	8 (3.39)	0 (0.00)	8 (4.02)	
Surgery(n,%)				
No	155 (65.68)	21 (56.76)	134 (67.34)	0.2909
Yes	81 (34.32)	16 (43.24)	65 (32.66)	
Diabetes(n,%)				
No	188 (79.66)	29 (78.38)	159 (79.90)	0.833
Yes	48 (20.34)	8 (21.62)	40 (20.10)	
CA19–9(n,%)				
≤37	59 (25.00)	9 (24.32)	50 (25.13)	0.918
>37	177 (75.00)	28 (75.68)	149 (74.87)	
CA125(n,%)				
≤35	51 (21.61)	6 (16.22)	45 (22.61)	0.5153
>35	185 (78.39)	31 (83.78)	154 (77.39)	
NAR(n,%)				
≤0.13	121 (51.27)	19 (51.35)	102 (51.26)	0.992
>0.13	115 (48.73)	18 (48.65)	97 (48.74)	
HALP(n,%)				
≤10	24 (10.17)	5 (13.51)	19 (9.55)	0.6623
>10	212 (89.83)	32 (86.49)	180 (90.45)	
Anti HBV treatment (n,%)				
No	216 (91.53)	17 (45.95)	199 (100.00)	<0.001
Yes	20 (8.47)	20 (54.05)	0 (0.00)	

**Figure 2:**
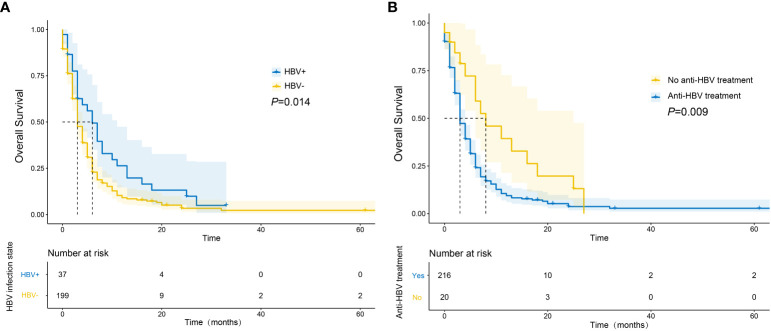
Survival curves of mPC patients with different HBV infection status **(A)** and whether they received anti-HBV drugs or not **(B)**.

A total of 206 deaths (87.3%) were recorded during the follow-up period. The median OS was significantly longer in the HBV-positive group compared to the HBV-negative group (6 vs. 3 months, *P* = 0.014; **Figure 2A**). Among HBV-positive patients, the median OS of those who had taken anti-HBV drugs was longer than that of those who had not (8 vs. 3 months, *P* = 0.009; **Figure 2B**).

### 3.2 Univariate and multivariate associations with OS

Using the X-tile, we determined the optimal cut-offs to be 0.13 for NAR and 10 for HALP. Univariate analysis identified age, sex, metastatic sites, chemotherapy, CA125, HALP, NAR, HBV infection status, and anti-HBV treatment as significantly associated with the prognosis of patients with mPC (**Table 2**). Multivariate analysis of Model 1 identified the following as independent risk factors for prognosis: HBV infection status (HR, 1.761 95% CI, 1.022–3.036, P = 0.042), sex (HR, 0.620, 95% CI, 0.463–0.831, P = 0.001), metastatic sites (HR, 1.572, 95% CI, 1.140–2.168, P = 0.006), chemotherapy (HR, 0.588, 95% CI, 0.436–0.793, P < 0.001), CA125 (HR, 2.158, 95% CI, 1.486–3.132, P <0.001), NAR (HR, 1.424, 95% CI, 1.071–1.894, P = 0.015) and HALP (HR, 0.536, 95% CI, 0.338–0.849, P =0.008).

**Table 2:** Univariate and multivariate Cox analysis for mPC patients.

Variable	Univariate		Multivariate(Model 1)		Multivariate(Model 2)	
	*HR*(95%*CI*)	*P*	*HR*(95%*CI*)	*P*	*HR*(95%*CI*)	*P*
Age						
≤60	Reference		Reference			
>60	1.465(1.111–1.933)	0.007	1.224(0.917–1.653)	0.171		
Sex						
Male	Reference		Reference		Reference	
Female	0.649(0.487–0.863)	0.003	0.620(0.463–0.831)	0.001	0.641(0.480–0.855)	0.002
Tumor location						
Head	Reference					
Body/Tail	1.094(0.819–1.462)	0.543				
Unknown	1.641(0.787–3.420)	0.186				
T stage						
T1	Reference					
T2	0.302(0.090–1.009)	0.052				
T3	0.496(0.154–1.601)	0.241				
T4	0.401(0.126–1.274)	0.121				
TX	0.508(0.147–1.758)	0.285				
N stage						
N0	Reference					
N1	0.984(0.674–1.437)	0.935				
N2	0.984(0.651–1.490)	0.941				
NX	0.933(0.464–1.879)	0.847				
Metastatic sites						
<3	Reference		Reference		Reference	
≥3	1.551(1.142–2.107)	0.005	1.572(1.140–2.168)	0.006	1.600(1.163–2.201)	0.004
Chemotherapy						
No	Reference		Reference		Reference	
Yes	0.740(0.561–0.976)	0.033	0.588(0.436–0.793)	<0.001	0.599(0.417–0.750)	<0.001
Radiotherapy						
No	Reference					
Yes	0.538(0.238–1.218)	0.137				
Surgery						
No	Reference					
Yes	0.815(0.607–1.094)	0.173				
Diabetes						
No	Reference					
Yes	1.071(0.762–1.504)	0.693				
CA19–9						
≤37	Reference					
>37	1.153(0.841–1.581)	0.376				
CA125						
≤35	Reference		Reference		Reference	
>35	1.856(1.311–2.627)	<0.001	2.158(1.486–3.132)	<0.001	2.299(1.588–3.330)	<0.001
NAR						
≤0.13	Reference		Reference		Reference	
>0.13	1.511(1.144–1.992)	0.004	1.424(1.071–1.894)	0.015	1.381(1.039–1.837)	0.026
HALP						
≤10	Reference		Reference		Reference	
>10	0.588(0.377–0.918)	0.019	0.536(0.338–0.849)	0.008	0.515(0.326–0.814)	0.004
HBV infection status						
HBV(+)	Reference		Reference		Reference	
HBV(-)	1.564(1.058–2.312)	0.025	1.761(1.022–3.036)	0.042	1.780(1.188–2.669)	0.005
Anti HBV treatment						
No	Reference		Reference			
Yes	0.526(0.309–0.893)	0.017	0.919(0.436–1.937)	0.832		

Similarly, Model 2 analysis of multivariate Cox regression showed that sex (P = 0.002), metastatic sites (P = 0.004), chemotherapy (P <0.001), CA125 (P <0.001), HALP (P = 0.004), NAR (P = 0.026) and HBV infection status (P =0.005) were also independent prognostic factors.

### 3.3 Development and validation of a predictive nomogram

**Figure 3:**
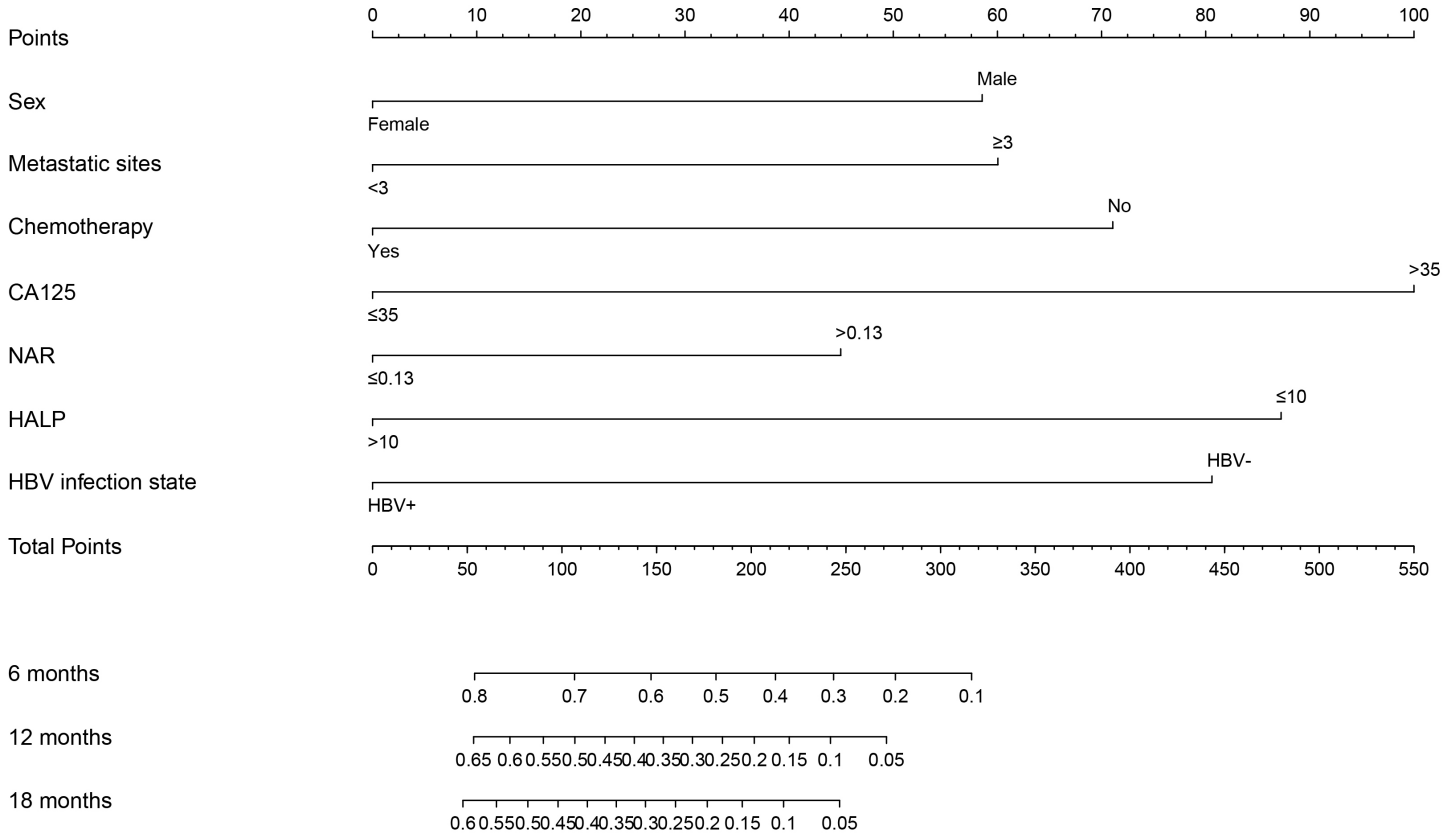
Nomogram of prognostic model for OS of mPC. Assessment of a nomogram based on seven predictors of overall survival (OS) in patients with metastatic pancreatic cancer.

**Figure 4:**
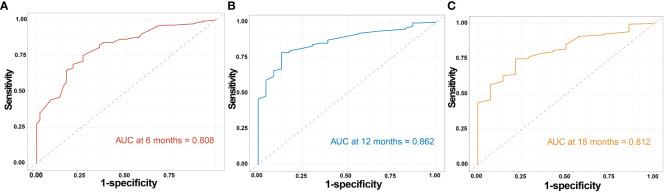
Time-dependent ROC curve of the model. Time-dependent receiver operating characteristic curves assessing the ability of the nomogram to predict overall survival (OS) at **(A)** 6 months, **(B)** 12 months, and **(C)** 18 months in metastatic pancreatic cancer.

**Figure 5:**
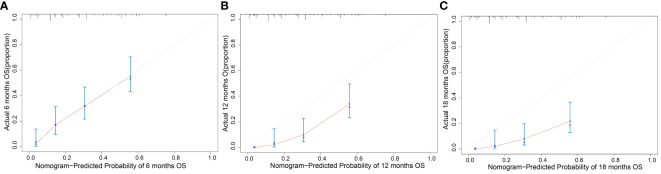
Calibration curve of the model. Calibration plot of the nomogram to predict overall survival (OS) at **(A)** 6 months, **(B)** 12 months, and **(C)** 18 months with metastatic pancreatic cancer.

**Figure 6:**
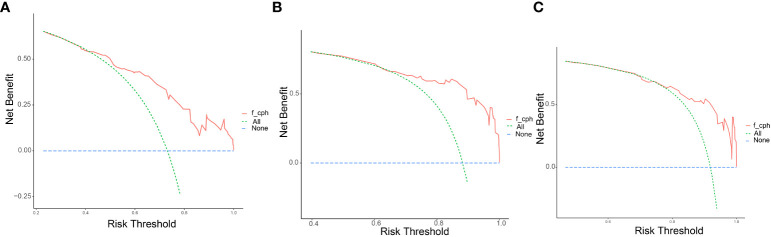
Decision curves of the model. Decision curve analysis (DCA) to assess the clinical benefit of the predictive nomogram at **(A)** 6 months, **(B)** 12 months, and **(C)** 18 months with metastatic pancreatic cancer.

The clinicopathological factors and HBV status identified in the multivariate analysis were used to construct a nomogram to predict the OS of patients with mPC at 6, 12, and 18 months (**Figure 3**). The nomogram showed that CA125, HALP, and HBV infection status were the most significant predictors of OS. Our nomogram demonstrated good discriminative power, with an AUC of 0.808 at 6 months, 0.862 at 12 months, and 0.812 at 18 months (**Figures 4A–C**). Calibration curves confirmed that the nomogram predicted prognosis most accurately at 6 months and less accurately at 12 and 18 months, these results were internally validated by bootstrapping (**Figure 5**). Nevertheless, decision curve analysis demonstrated that our nomogram could benefit patients at 6, 12, and 18 months after diagnosis (**Figure 6**).

### 3.4 Validation of the nomogram

**Figure 7:**
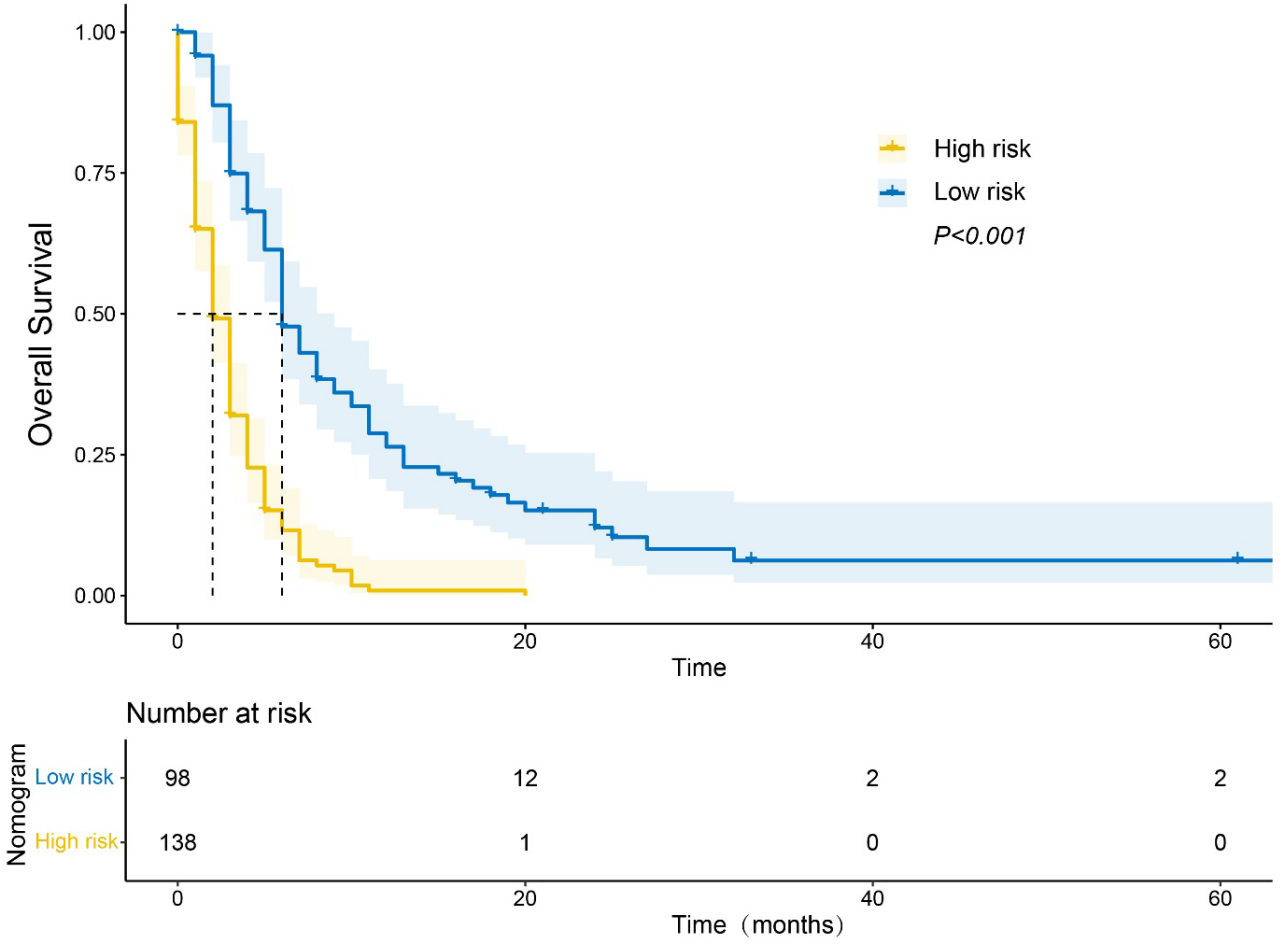
Kaplan−Meier curve of overall survival (OS) based on the nomogram. Notes: “Low-risk” patients were defined as those scoring < 270; other patients were classified as “high-risk”.

We assessed whether our nomogram accurately identified patients with mPC at different risk levels for poor OS. Based on the X-tile analysis, we determined a nomogram score of 270 to be the best cut-off for stratifying patients with mPC into those with short or long OS. Using this cut-off, patients were classified into a “low-risk” group with median OS of 6 months and a “high-risk” group with median OS of 2 months (P <0.001; **Figure 7**).

## 4 Discussion

Our study found that HBV infection was associated with better OS in patients with mPC. We also developed a nomogram based on HBV infection status, nutritional indicators, and clinicopathological characteristics, which may be useful for identifying patients with mPC at a higher risk of poor prognosis. This tool could guide the personalization of treatment and help allocate medical resources more efficiently.

Several studies have suggested that HBV infection contributes to the occurrence and progression of pancreatic cancer (11, 18, 19). However, in some European population studies, HBV infection was not found to be a risk factor for pancreatic cancer (20–22). Although the underlying mechanism by which HBV influences the development and progression of pancreatic cancer remains unclear, several possibilities exist. Firstly, HBV is a well-known carcinogen that is particularly prevalent in Asia, where it accounts for up to 80% of hepatocellular carcinoma cases (23). Secondly, while HBV is primarily hepatotropic and strongly associated with end-stage chronic liver disease (24), its systemic nature enables it to spread through the blood and deposit in organs other than the liver (25, 26). Given that the pancreas shares arteries and ducts with the liver, as well as progenitor cells originating from endoderm cells of the embryonic foregut, it could potentially serve as a target organ for HBV (27, 28). This provides a theoretical basis for the transfer of hepatitis virus to the pancreas. Once invaded by HBV, inflammation occurs in the pancreas. Chronic inflammation can damage pancreatic tissue, eventually leading to malignant transformation (29). Some studies have provided evidence of HBV infection markers in cancer tissues from patients with pancreatic cancer (29, 30). Batskikh et al. discovered the presence of HBV DNA and the expression of Hepatitis B virus X protein antigens in tissues of individuals with pancreatic cancer, demonstrating the replication ability of HBV within these tissues (7). Moreover, Hepatitis B virus X protein plays an important role in hepatocarcinogenic pathogenesis, and even without active replication, it can contribute to tumor cell transformation, invasion, and metastasis (31). Therefore, active replication of HBV within the pancreas may promote tumor cell transformation by infecting pancreatic cancer cells. HBV DNA can be integrated into the host genome, causing genomic mutations, enhancing the expression of proto-oncogenes, and reducing the relative expression of tumor suppressor genes, which may lead to tumorigenesis (31). Therefore, HBV may be involved in the development of non-hepatic tumors.

Surprisingly, we observed that HBV infection improved the prognosis of patients with mPC. Additionally, patients treated with anti-HBV drugs had a better prognosis. Future research should explore the underlying mechanisms behind this observation. However, the influence of HBV infection on the prognosis of pancreatic cancer remains controversial. For example, one study linked HBV to a heightened risk of synchronous liver metastases, thereby indicating a poorer prognosis (32). However, another study reported no significant disparity in short- or long-term survival between HBV-positive or -negative patients following pancreatic cancer resection (33). Such discrepancies may reflect variations in patient samples. Future research should explore the relationship between patients with mPC and OS through further large-sample and multicenter studies.

We hypothesized that HBV infection might improve the prognosis of patients with mPC through various mechanisms: Firstly, HBV has been shown to stimulate persistent inflammation by causing immune cells to release pro-inflammatory cytokines such as IL-6, IL-10, IL-12, TNF-α and IFN-γ (34–37). Most adult-onset HBV infections typically trigger robust, multifunctional CD8+ T cell responses (38). When HBV infects the host, HBV DNA replication may be initiated spontaneously or through active immunosuppression in some patients (39). Simultaneously, Hepatitis B virus X protein increases cyclooxygenase-2 expression, and HBV promotes immunosuppression by upregulating cyclooxygenase-2 (40, 41). In HBV-infected patients, circulating and tumor-infiltrating FoxP3^+^Tregs are enriched, exerting a suppressive effect on antitumor immunity (42). Therefore, we hypothesized that HBV regulates the pancreatic cancer tumor microenvironment by secreting these cytokines into pancreatic cancer cells, affecting the prognosis of patients with mPC. Moreover, the patients have been receiving regular anti-HBV treatment since hospitalization, and these drugs have demonstrated immunomodulatory effects (43). The immune response of CD8+T cells is enhanced in patients with chronic hepatitis B receiving anti-HBV therapy (44). Patients undergoing long-term nucleos(t)ide analogue therapy with low HBsAg levels may “wake up” dysfunctional immune cells due to reduced levels of viral antigen in the blood and liver (45). Even in HBV-infected patients, specific CD4+T cells play a crucial role in the sustained response after discontinuing anti-HBV drugs (46). This can influence the effectiveness of chemotherapy and the ability of the immune system to combat the tumor.

Several factors identified in our study as independent risk factors for poor OS in patients with mPC have also been associated with the prognosis of patients with pancreatic cancer following radical resection or those with incurable pancreatic cancer. These factors include high CA125, HALP, and NAR levels (14, 15, 47), aligning with existing literature, suggesting the reliability of our analyses and the resulting nomograms.

However, our findings should be interpreted cautiously in light of certain limitations, such as our small sample size and retrospective study design. These factors increase the risk of selection bias. Additionally, our sample spanned nearly a decade, during which the use and efficacy of antiviral treatments may have changed. Therefore, the generalizability of our findings should be verified and extended through multi-site prospective studies, which should also be used to externally validate our nomogram.

## 5 Conclusion

We constructed a nomogram based on the HBV infection status and nutritional-inflammatory biomarkers to predict the OS of patients with mPC. If externally validated, the nomogram could be a valuable tool for identifying high-risk patients and personalizing treatment strategies.

## Data availability statement

The data are not publicly available due to patient privacy. Requests to access the datasets should be directed to XLL, nllxl@163.com.

## Ethics statement

The studies involving humans were approved by the Ethics Review Committee of Guangxi Medical University Cancer Hospital (IRB approval number: LW2023006). The studies were conducted in accordance with the local legislation and institutional requirements. The participants provided their written informed consent to participate in this study. Written informed consent was obtained from the individual(s) for the publication of any potentially identifiable images or data included in this article.

## Author contributions

XWL: Conceptualization, Data curation, Formal analysis, Writing – original draft. QL: Software, Writing – original draft. SL: Data curation, Formal analysis, Writing – original draft. YL: Data curation, Formal analysis, Writing – original draft. XLL: Conceptualization, Resources, Supervision, Writing – review & editing.
